# A stochastically curtailed two-arm randomised phase II trial design for binary outcomes

**DOI:** 10.1002/pst.2067

**Published:** 2020-08-29

**Authors:** Martin Law, Michael J. Grayling, Adrian P. Mander

**Affiliations:** 1 Hub for Trials Methodology Research, Medical Research Council Biostatistics Unit, University of Cambridge, Cambridge, UK; 2 Population Health Sciences Institute, Newcastle University, Newcastle upon Tyne, UK; 3 College of Biomedical and Life Sciences, Cardiff University, Cardiff, UK

**Keywords:** adaptive design, cancer, continuous monitoring, interim analysis, oncology

## Abstract

Randomised controlled trials are considered the gold standard in trial design. However, phase II oncology trials with a binary outcome are often single-arm. Although a number of reasons exist for choosing a single-arm trial, the primary reason is that single-arm designs require fewer participants than their randomised equivalents. Therefore, the development of novel methodology that makes randomised designs more efficient is of value to the trials community. This article introduces a randomised two-arm binary outcome trial design that includes stochastic curtailment (SC), allowing for the possibility of stopping a trial before the final conclusions are known with certainty. In addition to SC, the proposed design involves the use of a randomised block design, which allows investigators to control the number of interim analyses. This approach is compared with existing designs that also use early stopping, through the use of a loss function comprised of a weighted sum of design characteristics. Comparisons are also made using an example from a real trial. The comparisons show that for many possible loss functions, the proposed design is superior to existing designs. Further, the proposed design may be more practical, by allowing a flexible number of interim analyses. One existing design produces superior design realisations when the anticipated response rate is low. However, when using this design, the probability of rejecting the null hypothesis is sensitive to misspecification of the null response rate. Therefore, when considering randomised designs in phase II, we recommend the proposed approach be preferred over other sequential designs.

## 1 Introduction

Single-arm binary outcome phase II trials require fewer participants than equivalent randomised two-arm trials, making single-arm trials a pragmatic choice in many instances. The data from single-arm binary outcome trials are typically compared to a pre-specified historical control response rate. However, this comparison may not be valid,^1,2,3^ given, for example, that a systematic review of phase II oncology trials found that 46% of the reviewed trials did not cite a source for the historical response rate used.^1^ Two-arm randomised trials directly compare the responses of two groups from the same population, where one group has been allocated to treatment and the other group has been allocated to control. This is preferable to a non-randomised trial comparing the responses from a contemporary population to those from a historical population, which may differ in characteristics. Although single-arm trials are more common in small populations and in rare disease settings, randomised designs should still be preferred when at all possible, as using historical information may provide less robust evidence.^4,5^ Thus, it has been argued that in almost all instances, two-arm randomised trials should be preferred to single-arm trials, with two-arm randomised trials considered to be the gold standard in trial design.^6,7^ Nevertheless, single-arm trials remain popular in phase II oncology, accounting for 57% of trials in a recent review of 557 trials.^8^ It is, therefore, of interest to reduce sample sizes in two-arm phase II binary outcome trials, so that it may become possible to use two-arm designs when previously only a single-arm design would be considered, either due to cost or recruitment difficulties. For further details on the choice of single-arm or randomised designs, see Grayling et al.^2^

One approach to reducing sample size is to allow early stopping in the form of interim analyses. In the area of single-arm trials, the most frequently implemented such design is by Simon,^9^ where, at a single interim analysis, the trial may stop early due to a lack of response. An analogous two-arm design has been proposed by Jung^10^; as with Simon, there is a single interim analysis and the trial may end at this point due to a lack of response. In this case, a lack of response entails observing a low response rate on the experimental treatment arm compared to the control arm.

Further sample size savings can be made by allowing a trial to stop as soon as the final trial decision is certain, known as non-stochastic curtailment (NSC), with respect to either a positive effect or lack of positive effect on the treatment arm compared to the control arm. This can be incorporated as an extension of Jung’s design,^10^ by additionally allowing the trial to stop immediately if the response rates are such that the final decision to reject or not reject the null is known with certainty, either at the interim (to not reject only) or by the end (to either reject or not reject). This approach was first proposed by Carsten and Chen.^11^ Their proposed design allows stopping after every pair of participants, where each pair is allocated evenly to experimental treatment and control, therefore assuming perfect balance. As in Jung’s design, an interim stopping boundary is used, which allows stopping only for a lack of response. “Success” is determined for every (balanced) pair of results, where success is defined as a pair of results where response on treatment and non-response on control is observed; all other combinations of results are treated equally, as a non-success. The test statistic is then the total number of such successes. The trial stops as soon as a pre-specified required number of successes is observed, or as soon as the number of successes required (either at the interim or at the end of the trial) cannot be reached. The incorporation of NSC into Jung’s design is also proposed by Chen et al,^12^ where the proposed design allows stopping after every patient. In this design, success is determined for each patient and is defined as a response for a participant on treatment or a non-response for a participant on control. The test statistic in this case is the difference in the number of responses between the treatment and control arms; the same test statistic as is used by Jung.^10^ The trial stops as soon as the required difference in the number of responses is guaranteed to be reached, or cannot be reached (again, either at the interim or by the end of the trial). Thus the designs of Carsten and Chen and Chen et al require continuous or almost continuous monitoring.^11,12^

We present a design approach that produces two-arm trials with expected sample sizes that are closer to those of single-arm trials than to typical two-arm trials. This design permits early stopping of a trial not only when reaching or failing to reach the required difference in the number of responses is certain but also when it is very likely, known as stochastic curtailment (SC). The rationale behind SC is that when the probability of success given current results is great, for example, 0.99, it makes sense to end that trial if a consequence is a reduction in sample size. SC has previously been applied to single-arm trials.^13,14,15^ However, SC has not previously been applied to two-arm trials. Another clinical trial characteristic that is utilised in our approach is block randomisation. In block randomisation, a block size is chosen and randomisation takes place within blocks such that allocation is equal between the experimental treatment and control arms. This places an upper bound on the degree of allocation imbalance that may occur. We introduce an approach to designing a block randomised, two-arm binary outcome trial that uses SC, where stopping is permitted after each block. Permitting stopping after each block is a sensible approach to early stopping, allowing trials to end early but without the need to make a decision after every participant or pair of participants, which may be impractical in large randomised two-arm trials. Further, the block size may be chosen to suit the resources of the trial, with smaller block sizes allowing decisions to be made earlier and larger block sizes requiring fewer early stopping decisions. It may be possible to undertake continuous monitoring and update a trial design after each participant.^16^ However, in some circumstances, this is not possible, and the proposed design does not require such a degree of monitoring. The flexible framework permits a wide range of degrees of monitoring, from a small number of interim analyses to monitoring that is almost continuous.

We note that other two-arm approaches have been proposed.^17,18,19,20,21,22^ However, it is impractical to compare all randomised two-arm designs, and so our approach will be compared only to Jung’s design, as this is the two-arm analogue to the popular Simon design, and Carsten and Chen and Chen et al’s designs, as these designs use curtailment and as such are similar to our approach.^10,11,12^ Table 1 shows the main differences between the designs to be compared. As can be seen, our approach uses a test statistic that has been used in other approaches, allows both stochastic and NSC, allows early stopping solely based on how likely trial success is, and allows a flexible number of interim analyses.

In the Methods section, we explain the proposed approach, how design realisations are found and how the approach will be compared to existing designs. In the Results section, our approach is compared to those of Jung, Carsten and Chen and Chen et al.^10,11,12^ These comparisons are undertaken in terms of a loss function in the form of a weighted sum of optimality criteria, as well as through sensitivity to misspecification of response rates and a real-life example. The Discussion section puts the results into context with respect to existing designs.

## 2 Methods

For a two-arm trial, let the true response rate on the control and treatment arms be *p_C_* and *p_T_*, respectively. The nature of hypothesis testing requires us to consider what difference in treatment effect between the two arms is worth further study. To this end, we let *p*_0_ and *p*_1_ be the anticipated response rates in the control and treatment arms respectively, such that the treatment difference *p*_1_ − *p*_0_ is worth further study. Our null hypothesis is as follows: *H*_0_ : *p_T_* ≤ *p_C_*. Denoting by P(reject *H*_0_ | *p_C_*, *p_T_*) the probability that *H*_0_ is rejected given response rates *p_C_* and *p_T_*, our design will guarantee P(reject *H*_0_ | *p*_0_, *p*_0_) ≤ *α* and P(reject *H*_0_ | *p*_0_, *p*_1_) ≥1 − *β* for specified error rates *α* and *β*.

For the proposed approach, a balanced allocation between treatment and control, that is, 1:1 randomisation, is sought. The design involves frequent interim analyses, at most after every pair of observed results (one each on the control and treatment arms). Let N be the maximum possible total sample size and let the number of participants so far on the control and treatment arms be *m_C_* and *m_T_*, respectively. Using randomised blocks and undertaking, an interim analysis only at the end of each block ensures balance across the two arms at each analysis. As such, let *m* be the number of participants so far on each arm, for *m* = *B*, 2*B*, …, *N*/2, where 2*B* is the number of participants in each block. Let *X_C_*(*m*) and *X_T_*(*m*) be the number of binary responses on the control and treatment arms after *m* participants on each arm. Within each block, the number of responses on each arm follows the binomial distributions *X_C_*(*B*) ~ *Binom*(*B*, *p_C_*) and *X_T_*(*B*) ~ *Binom*(*B*, *p_T_*), for a fixed number of participants *B* per block per arm. The test statistic we will use to determine whether to reject the null hypothesis is the difference in the number of responses between the arms, *X_T_*(*m*) − *X_c_*(*m*), though other valid test statistics exist. In particular, the decision of whether to study a treatment further may depend not solely on rejection of the null hypothesis, but also on other factors. For example, a design may explicitly require a minimum effect size estimate before permitting further study.^23,24,25^ Some such methods involve sculpting of the rejection region. However, the true response rates *p_C_* and *p_T_* may not be equal to the anticipated response rates *p*_0_ and *p*_1_, that is, the response rates may be misspecified. If so, sculpting the rejection region can lead to underestimating the type I error.^26^ Sensitivity to such misspecification will be examined in the Results section and we discuss this issue further in the Discussion. Moreover, it is usually possible to design a trial by specifying the improvement in response rate that would be clinically worthwhile.^7^

### 2.1 Curtailment

At each interim analysis, there are three possible courses of action: stop the trial to make a go decision and reject the null hypothesis; stop the trial to make a no go decision and do not reject the null hypothesis, or continue recruitment. Define success as observing a response on the treatment arm or a non-response on the control arm, with the number of successes in a trial so far defined as *S*(*m*) ≔ *X_T_*(*m*) + *m* − *X_C_*(*m*). The course of action taken is determined by comparing the number of successes so far to some specified lower and upper boundaries, the calculation of which is described below. For the final analysis, it is not possible to continue the trial further, therefore either a go or no go decision is made and only a single boundary is required. Let this final stopping boundary be *r*. A go decision, that is, a decision to reject *H*_0_, is made at an interim analysis if the final difference in number of responses is guaranteed to be *r* or greater in favour of the treatment arm. This occurs at the end of the trial if *X_T_*(*N*/2) − *X_c_*(*N*/2) ≥ *r* or before the end of a trial if *X_T_*(*m*) + *m* − *X_c_*(*m*) ≥ *N*/2 + *r*. A no go decision, that is, a decision to not reject *H*_0_, is made as soon as the final difference in responses is guaranteed to not be *r* or greater in favour of the treatment arm; this occurs before the end of a trial if *m* - *X_T_*(*m*) + *X_c_*(*m*) ≥ *N*/2 − *r* + 1. These decision rules are the NSC boundaries used by Chen et al,^12^ though we relax their requirement for continuous monitoring. Jung, Carsten and Chen and Chen et al also include an explicit interim analysis, that is, an interim analysis at which point a go/no go decision is made regardless of whether or not the final pre-specified stopping boundary may be reached.^10,11,12^ However, such an approach may result in a no go decision even when there is a high probability of correctly identifying that the null hypothesis is false. This issue has been highlighted previously for trials using Simon’s design, for example, a Simon design used in a study by Sharma et al contained a no go stopping boundary at a point where the probability of correctly identifying a treatment with the desired response rate was 0.8.^15,27^ Therefore, the proposed design avoids this issue by not including such an interim analysis. While SC, a feature of our proposed design, by definition permits early stopping without knowing with certainty whether the final stopping boundary will be reached, the proposed design quantifies at each interim the probability of an efficacious treatment reaching the final stopping boundary. Further, lower and upper bounds for this probability can be set by the investigator.

### 2.2 Conditional power

The conditional probability, *CP*(*p_C_*, *p_T_*, *S*, *m*), is the probability of rejecting *H*_0_ conditional on observing *S* successes after *m* participants on each arm assuming some response rates *p_C_* and *p_T_*, with *r* and *N* fixed. Setting *p_C_* = *p*_0_, *p_T_* = *p*_1_ gives the conditional power *CP*(*p*_0_, *p*_1_, *S*, *m*). For the purposes of the proposed approach, the only conditional probability of interest is the conditional power, and so *CP*(*S*, *m*) will subsequently refer solely to conditional power *CP*(*p*_0_, *p*_1_, *S*, *m*), while the abbreviation CP will refer to conditional power in general. *CP*(*S*, *m*) is calculated using *p*_0_ and *p*_1_, that is, there is no re-estimation of response rates. SC in this design is based on CP, though we acknowledge that other approaches are available.^28^

#### 2.2.1 Calculating conditional power under NSC

When *m* − *X_T_*(*m*) + *X_C_*(*m*) ≥ *N*/2 − *r* + 1, it is no longer possible for the null hypothesis to be rejected, and so *CP*(*S*, *m*) is equal to zero. Conversely, when *X_T_*(*m*) + *m* − *X_C_*(*m*) ≥ *N*/2 + *r*, rejection of the null hypothesis is guaranteed, and so *CP*(*S*, *m*) is equal to one. For a block design with no explicit interim analysis and using NSC but not SC, *CP*(*S*, *m*) can be written recursively as

(1) $$CP(S,m)=\left\{\begin{array}{ll} 0, & \text{if}\;m-X_{T}(m)+X_{C}(m)\geq N/2-r+1\\ D, & \text{if}\;m-X_{T}(m)+X_{C}(m)<N/2-r+1\;\text{and}\;X_{T}(m)+m-X_{C}(m)<N/2+r\\ 1, & \text{if}\;X_{T}(m)+m-X_{C}(m)\geq N/2+r \end{array}\right\},$$

where

$$D=\sum_{i=0}^{2B}P\left(i,B|p_{0},p_{1}\right)CP(S+i,m+B)$$

and P(*i*, *B* | *p_C_*, *p_T_*) = *P*(*S*(*B*)=i| *p_C_*, *p_T_*) = *P*(*X_T_*(*B*) + *B* − *X_C_*(*B*) = *i*| *p_C_*, *p_T_*), the probability of observing *i* successes from a block containing *B* participants on each arm, given response rates *p_C_* and *p_T_*.

#### 2.2.2 Calculating conditional power under SC

SC entails ending a trial not only at any point where CP is equal to zero or one, but also for a no go decision at any point where 0 < *CP*(*S*, *m*) < *θ_F_* or for a go decision at any point where *θ_E_* < *CP*(*S*, *m*) < 1, for fixed thresholds (*θ_F_*, *θ_E_*) ∈ [0, 1] such that *θ_F_* < *θ_E_*. The subscripts *F* and *E* have been chosen as a no go decision is typically associated with treatment futility and a go decision with treatment efficacy.

To incorporate SC, only slight changes to Equation (1) are required:

(2) $$CP(S,m)=\left\{\begin{array}{ll} 0, & \text{if}\;m-X_{T}(m)+X_{C}(m)\geq N/2-r+1\;\text{or}\;D<\theta_{F}\\ D, & \text{if}\;m-X_{T}(m)+X_{C}(m)<N/2-r+1\;\text{and}\;X_{T}(m)+m-X_{C}(m)<N/2+r\;\text{and}\;\theta_{F}\leq D\leq \theta_{E}\\ 1, & \text{if}\;X_{T}(m)+m-X_{C}(m)\geq N/2+r\;\text{or}\;D>\theta_{E} \end{array}\right\},$$

Equations (1) and (2) are recursive as the CP at a given point, *CP*(*S*, *m*) say, is dependent on the CP at “future” points *CP*(*S* + *i*, *m* + *B*), *i* = 0, 1…, 2*B*. Under SC (Equation (2)), CP values lower than *θ_F_* are rounded down to zero and CP values greater than *θ_E_* are rounded up to one, as early stopping occurs at such points. These equations are analogous to the calculation of CP in single-arm trials introduced by Law et al.^15^ Calculating CP in this manner accounts for the possibility of early stopping due to SC. Thus it ensures that the operating characteristics are known exactly, and that any decision to continue the trial is done so knowing exactly what degree of uncertainty remains about whether to reject *H*_0_. By calculating the CP at each point (*S*, *m*), *m* = *B*, 2*B*, …, N/2, *S* =0, 1, …, 2*m*, the stopping boundaries for the conclusion of each block are obtained. Knowing in advance which points will, if reached, result in early stopping means that the exact distribution of the trial’s outcomes are known. Further, calculating CP without error at each point, rather than using an approximation, prevents decisions being made based on a CP with unknown error.

Let any particular example of a trial created using our approach be characterised by (*r*, *N*, *B*, *θ_F_*, *θ_E_*, *p*_0_, *p*_1_), and denote any such example to be a “realisation” of our design. Each design realisation has explicit lower and upper bounds for CP, *θ_F_* and *θ_E_*, one of which must be reached before the trial may end. For existing design approaches that permit early stopping to reject *H*_0_ under NSC only, such as Carsten and Chen and Chen et al, the upper bound for rejecting the null hypothesis in terms of CP is always fixed at *θ_E_* = 1.^11,12^ That is, the CP must be equal to one in order to reject the null hypothesis, and this value may not be altered. Further, the designs of Jung, Carsten and Chen and Chen et al include an explicit interim analysis, containing some stopping boundary that does not take into account the probability of reaching the final stopping boundary. Consequently, existing designs have no fixed lower bound *θ_F_* for ending the trial to make a no go decision; the trial is ended simply if it is no longer possible to reach the interim stopping boundary. Thus for these existing designs, with regards to stopping at or prior to the interim analysis, the CP is not evaluated and hence not known. This may lead to making a no go decision when the CP is high, as described above.

### 2.3 Search for admissible designs

The paramount requirements of a design realisation are that the desired type I error *α* and power 1 − *β* are satisfied, that is, α* = P(reject *H*_0_| *p*_0_, *p*_0_) ≤ *α* and 1 − β* = P(reject *H*_0_| *p*_0_, *p*_1_) ≥ 1 − *β*, where α* and 1 − β* are characteristics of a given design realisation. Designs that satisfy these requirements are denoted *feasible*. We wish to consider only feasible designs. It is worthwhile to compare the expected sample size of feasible design realisations. Let the expected sample size for response rates *p_C_* and *p_T_* on control and treatment arms respectively be *ESS*(*p_C_*, *p_T_*). Expected sample size for a given design is obtained by finding all possible points at which the trial will end, then multiplying the number of participants so far at those points by the probability of reaching such points:

$$ESS\left(p_{C},p_{T}\right)=\sum_{j=1}^{N/2B}\sum_{i=0}^{2jB}2jB\;P\left(i,jB|p_{C},p_{T}\right)I(CP(i,jB)\in \{0,1\}).$$

Our interest lies in *ESS*(*p*_0_, *p*_0_) and *ESS*(*p*_0_, *p*_1_). When searching for designs, we exclude design realisations that are not superior to others with respect to at least one of the following criteria: expected sample size under *p_C_* = *p_T_* = *p*_0_, *ESS*(*p*_0_, *p*_0_), expected sample size under *p_C_* = *p*_0_, *p_T_* = *p*_1_, *ESS*(*p*_0_, *p*_1_) or maximum sample size *N*. Such excluded designs are termed *dominated*. Design realisations that are both feasible and not dominated have previously been described as *admissible* designs,^10,29^ and these designs are our subject of interest. It is the admissible designs of our proposed approach that will be compared, both to one another and to admissible designs of other approaches.

In order to find admissible designs, a search of possible designs is undertaken. The block size 2*B*, desired type I error *α* and power 1 − *β* are specified in advance, as is an upper bound for maximum sample size, *max*(*N*), as may a range for the final stopping boundary *r*. Also specified in advance are a maximum lower bound and minimum upper bound for CP, denoted *θ_F_MAX__* and *θ_E_MIN__*, so that the design search takes place only over combinations (*r*, *N*, *B*, *θ_F_*, *θ_E_*) that satisfy *θ_F_* ≤ *θ_F_MAX__* and *θ_E_*≥*θ_E_MIN__*, as per Law et al.^15^ For all results that follow, *θ_F_MAX__* was set equal to *p*_1_, that is, a trial’s CP threshold for ending for a no go decision may not be greater than the anticipated response rate on treatment, or *θ_F_* ≤ *p*_1_. This is a pragmatic choice: it is a sensible constraint to not consider a no-go decision if the current conditional probability of trial succ*ESS* is greater than the probability of observing a response in a single participant allocated to a treatment with response rate *p*_1_. A fixed value of 0.7 was chosen for *θ_E_MIN__*, meaning that a trial’s CP threshold for ending for a go decision may not be less than 0.7, that is, *θ_E_* ≥ 0.7. This value was considered a reasonable minimum probability for making a go decision, though in practice this value may be determined in collaboration with investigators. If an investigator wishes to allow early stopping for a go decision only when CP is high, then this may be set to, for example, *θ_E_MIN__* =0.95, or even *θ_E_MIN__* = 1 if an investigator wishes to permit early stopping for a go decision only when reaching the final stopping boundary *r* is certain. The final value that may be specified is the maximum number of (*θ_F_*, *θ_E_*) combinations to be tested per unique (*r*, *N*). This is further explained below.

Searches were undertaken for two block sizes, 2*B* ∈ {2, 8}, and for five values of control arm response rate, *p*_0_ ∈ {0.1,0.2,0.3,0.4,0.5}, with *p*_1_ = *p*_0_ + 0.2 in each instance. This resulted in 10 searches overall. These block sizes were chosen to examine to what extent the operating characteristics change when the degree of monitoring is reduced considerably. The searches had the following parameters: *max*(*N*) = 120, *r* ∈ {0, 1, …, ⌈*N*_p__1_⌉}, *α* = 0.15, *β* = 0.2 (as in Table 1 of Jung^10^), *θ_F_MAX__* = *p*_1_, *θ_E_MIN__* = 0.7 and maximum number of (*θ_F_*, *θ_E_*) combinations 10^6^. The maximum sample size, CP thresholds and maximum number of (*θ_F_*, *θ_E_*) combinations were pragmatic choices, balancing the desire to search over as many design realisations as possible against computational intensity. Each trial, with design parameters (*r*, *N*, *B*, *θ_F_*, *θ_E_*, *p*_0_, *p*_1_), was evaluated to obtain α*, β*, *ESS*(*p*_0_, *p*_0_) and *ESS*(*p*_0_, *p*_1_).

#### 2.3.1 Searching over CP thresholds *θ_F_* and *θ_E_*

For any given trial design (*r*, *N*, *B*), each possible combination of successes, *S*, and participants so far, 2*m*, has an associated CP. As *θ_F_* and *θ_E_* vary, the operating characteristics of a trial are only certain to change when *θ_F_* or *θ_E_* become greater than or less than one of the possible CP values in the trial. As such, we have chosen to vary *θ_F_* and *θ_E_* over the trial-specific CP values rather than searching over uniform distributions of *θ_F_* and *θ_E_*. That is, (*θ_F_*, *θ_E_*) ∈ {*CP*(*S*, *m*)}, *m* = *B*, 2*B*, …, *N*/2, *S* =0, …, 2*m*, s.t. *θ*_F_ < *θ_E_*,*θ_F_* ≤ *θ_F_MAX__*, *θ_E_*≥*θ_E_MIN__*.

For large sample sizes, the number of unique CP values and consequently, the number of possible (*θ_F_*, *θ_E_*) combinations to be searched over may be great. However, certain aspects of our design approach can ameliorate this to some degree. Firstly, in many cases, the CP is equal to zero or one. Secondly, by only permitting stopping after every 2*B* participants, we need only consider the CP values that occur at the conclusion of each block, that is, after *B*, *2B*, …, *N*/2 participants on each arm. Finally, there are the user-defined limits set above: *θ_F_* ≤ *θ_F_MAX__*, *θ_E_* ≥*θ_E_MIN__*. These three aspects reduce the number of unique CP values for each trial design. Nevertheless, the number of possible (*θ_F_*, *θ_E_*) combinations still increases rapidly with N. As such, a limit may be imposed on the maximum number of combinations that may be examined per (*r*, *N*, *B*). As stated above, the limit chosen was 10^6^, meaning that for each (*r*, *N*, *B*) combination, at most 10^6^ combinations of (*θ_F_*, *θ_E_*) are examined. When there are more than 10^6^ possible combinations, the CP values are ordered from smallest to greatest, then every other value is removed. This halving repeats until the number of possible combinations remaining is not greater than 10^6^. The same approach is undertaken in Law et al.^15^ As the distribution of CP values in a trial is not uniform,^15^ this approach is less likely to miss potential designs than simply searching over a uniform distribution of CP values.

Regarding searches using existing designs, admissible designs for Jung’s design were found using the R package ph2rand.^10,30^ Admissible designs for the designs of Carsten and Chen and Chen et al were found using simulation as suitable code was not available: all design combinations (*r_1_*, *n_1_*, *r*, *N*) such that *N* ∈ [10,200], with restrictions r_1_ ≤ *n*_1_, *r*_1_ ≤ *r*, were obtained, where *r*_1_ denotes the interim stopping boundary that must be reached after *n*_1_ participants for the trial to continue.^11,12^ For each design, α* and β* were initially estimated using 100 simulated datasets. Designs with α* > 0.25 (ie, *α* + 0.1) or β* > 0.3 (ie, *β* + 0.1) were discarded. For the remaining designs, α*, β*, *ESS*(*p*_0_, *p*_0_) and *ESS* (*p*_0_, *p*_1_) were estimated using 10 000 simulated datasets. Designs with α* > 0.15 or β* > 0.2 were discarded, as were dominated designs, leaving a set of admissible designs for both approaches. To avoid confusion, the design of Carsten and Chen will be described in the Results section as “Carsten”.^11^

The code to undertake the design search for the proposed design was written in R.^31^ It is available in the supplementary material and on github.^32^

### 2.4 The loss function

The concept of using a loss score in the form of a weighted sum of optimality criteria to compare trial designs has been used previously.^10,15,29^ We use the approach of Mander et al,^29^ in which the loss score of a design is defined as

$$L=w_{0}ESS\left(p_{0},p_{0}\right)+w_{1}ESS\left(p_{0},p_{1}\right)+\left(1-w_{0}-w_{1}\right)N,$$

where *w*_0_, *w*_1_ ∈ [0, 1] and *w*_0_ + *w*_1_ ≤ 1. For all combinations of weights *w*_0_ and *w*_1_, the loss score is compared across admissible designs from different approaches. To further compare admissible designs produced by different approaches, we use the method of Law et al of noting the design realisation with the lowest loss score for each combination of weights (among all design approaches).^15^ This design is termed the omni-admissible design. The omni-admissible design is deemed to be the best-performing design realisation for that combination of weights. The design type of each omni-admissible design is obtained for each combination of weights and the results plotted, to visualise what approach performs best for each weighting of optimality criteria.

## 3 Results

### 3.1 Comparing design approaches using multiple criteria

All results are based on the operating characteristics (*α*, *β*) = (0.15,0.20), as used in table 1 of Jung,^10^ and the range *p*_0_ = 0.1, …, 0.5, *p*_1_ = *p*_0_ + 0.2. To address the case of greater response rates, a real-life example is investigated where *p*_0_ = 0.70, *p*_1_ = 0.85.

**Figure 1:**
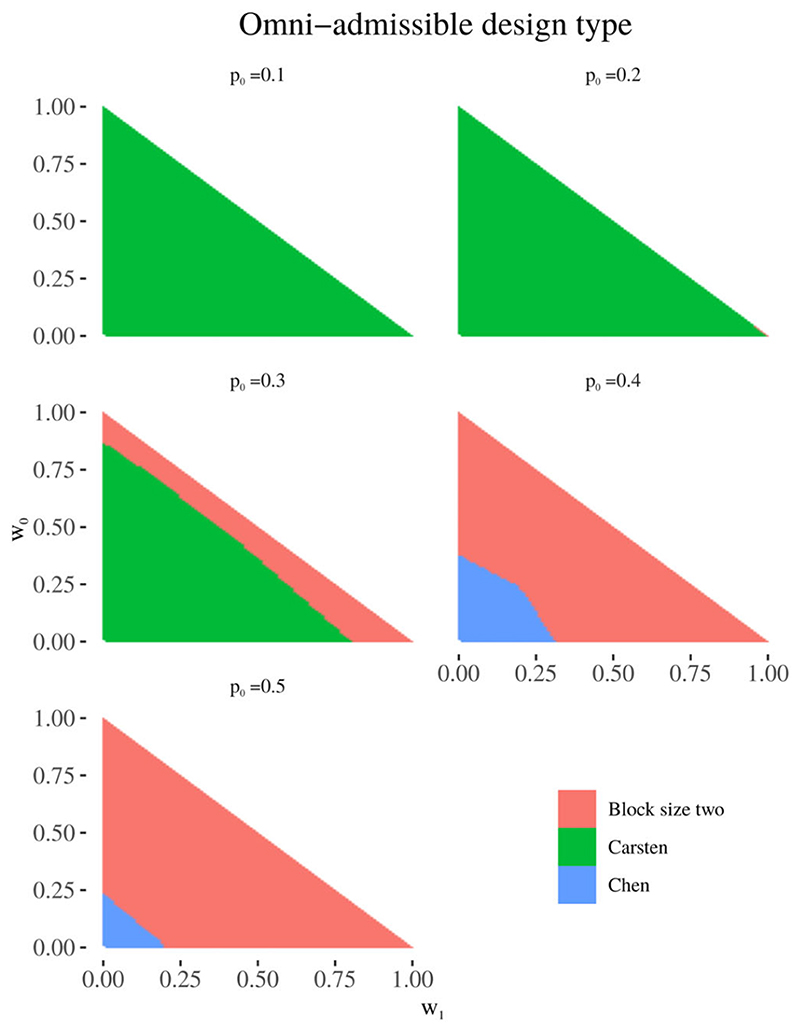
Omni-admissible design: the approach to which the design realisation with the lowest loss score belongs, for (α, β) = (0.15, 0.2), p0 = 0.1, 0.2, 0.3, 0.4, 0.5, p1 = p0 + 0.2

Figure 1 shows the design approach to which the omni-admissible design belongs, that is, the design with the lowest loss score among those compared, for all combinations of weights (*w*_0_, *w*_1_). For *p*_0_ = 0.1 and *p*_0_ = 0.2, Carsten’s design is superior in almost all instances (100% of weights for *p*_0_ = 0.1, 99% for *p*_0_ = 0.2). For *p*_0_ = 0.3, the omni-admissible design is either a Carsten design (73%) or a block design with block size two (27%). For *p*_0_ = 0.4 and *p*_0_ = 0.5, the omni-admissible design is either a block design with block size two or Chen’s design (85% vs 15% for *p*_0_ = 0.4, 95% vs 5% for *p*_0_ = 0.5). There are no regions for which the omni-admissible design belongs to Jung’s design, as the approach of Chen et al can be considered to produce design realisations encompassing all possible Jung design realisations but with the addition of NSC.^10,12^ There are also no regions for which the omni-admissible design belongs to our approach with block size eight, however this may be expected as any design using blocks of size eight will generally be outperformed by the equivalent design with blocks of size two.

**Figure 2:**
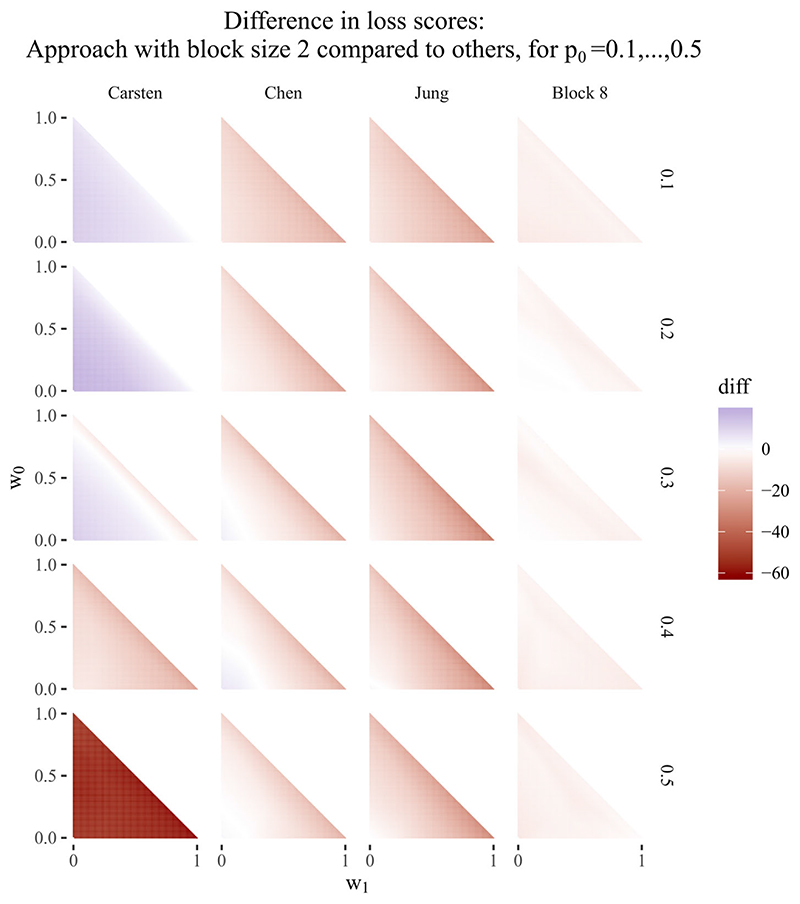
Difference in loss scores for block design of size two vs other approaches, for p_0_ = 0.1, …, 0.5. Negative values (in red) favour block design

**Figure 3:**
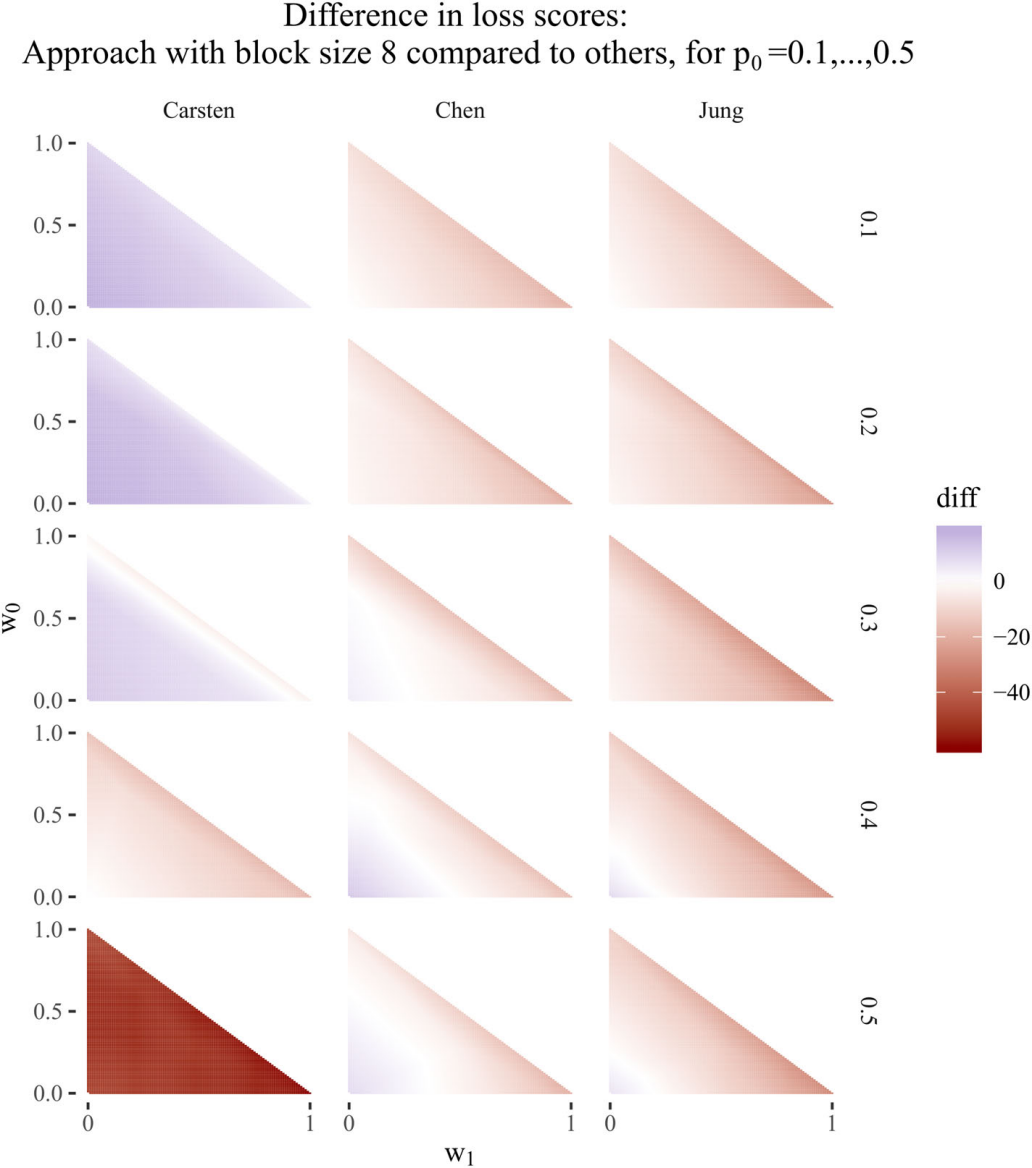
Difference in loss scores for block design of size eight vs other approaches, for p_0_ = 0.1, …, 0.5. Negative values (in red) favour block design

Figure 2 shows the difference in loss scores between the block design using block size two, existing designs and block size eight, again for all possible weights. The difference is taken between the design realisations with the lowest loss scores for a given weight combination, ensuring that the best design realisation for each design approach is being compared. As with Figure 1, the plots show that while Carsten’s design is superior to the block approach for low values of *p*_0_, it performs comparatively less well as *p*_0_ increases. This result was also found by Chen et al,^12^ and can be seen particularly on the bottom row of plots in Figure 2, where Carsten’s designs perform poorly in comparison to the other designs. The rightmost column of plots compares block size two to block size eight, and is close to white at all points, showing that the difference between the designs is always close to zero in terms of loss score. The maximum difference in loss score in favour of block size two compared to block size eight is six units across all combinations of weights, compared to 61 units for block size two compared to Carsten, 25 units compared to Chen et al and 35 units compared to Jung. An equivalent set of plots, comparing block size eight to existing designs, is shown in Figure 3, and shows similar results: both plots show the superiority of the proposed approach compared to existing designs, even when monitoring is reduced to conducting an interim analysis only after each block of eight participants.

As an example of the design realisations produced by each approach, Table 2 shows a selection of design realisations, including the Simon design, for the set of design parameters (*α*, *β*, *p*_0_, *p*_1_) = (0.15,0.20,0.30,0.50), *p*_0_ = 0.3 being the midpoint of the five values chosen for *p*_0_. For each design, the table shows the design realisations that minimise *ESS*(*p*_0_, *p*_0_) and *ESS*(*p*_0_, *p*_1_) (the *p*_0_- and *p*_1_-optimal designs respectively), as well as those with the lowest *ESS*(*p*_0_, *p*_0_) and *ESS*(*p*_0_, *p*_1_) among the designs with the minimum maximum sample size *N* (the *p*_0_- and *p*_1_-minimax designs, respectively). In this case, the *p*_0_- and *p*_1_-minimax designs are identical for all designs considered. All designs that use curtailment are superior to Jung’s design in each of the criteria of interest (*p*_0/1_-optimal and *p*_0/1_-minimax).

For the *p*_0_-optimal designs, the block designs achieve lower expected sample size *ESS*(*p*_0_, *p*_0_) than the existing randomised designs (47.3, 49.2 vs 64.9, 51.3, 60.1) at the expense ofgreater maximum sample size *N* (116, 112 vs 92, 88, 90). This is also the case for the *p*_1_-optimal designs with regards to *ESS*(*p*_0_, *p*_1_) (45.4, 49.3 vs 80.8, 52.6, 67.3, *N*=112, 112 vs 82, 92, 76). The Simon designs have expected sample sizes in the interval [17.0,25.1], while the standard two-arm trial with no early stopping has expected sample size *ESS*(*N*) = *N* = 124. As such, *ESS*(*p*_0_, *p*_0_) and *ESS*(*p*_0_, *p*_1_) for both block designs are closer to those of the Simon design than the two-arm sample size, under all optimality criteria.

The cases where existing two-arm designs are superior to the proposed block designs under the criterion being minimised are the Carsten and Chen et al designs under *p*_0/1_-minimax, where these designs achieve a lower maximum sample size compared to the block designs (68, 76 vs 80, 80). However, the block design with block size eight requires less monitoring than the existing designs, with a maximum of 10 decisions compared to 34 decisions for Carsten and 76 for Chen et al.^11,12^ These comparisons of maximum and expected sample sizes are reflected in the left-hand plots of the third row of Figure 2, where the triangle is red near the hypotenuse, indicating superiority of the block design when minimising expected sample size is of greatest value, and the triangle is blue near the lower left corner, indicating superiority of Carsten’s and Chen et al’s designs when minimising maximum sample size is of greatest value.

To address possible concerns regarding very early stopping, the minimum possible number of participants was obtained for the *p*_0/1_-optimal and *p*_0/1_-minimax block designs. The median minimum number of participants for block size two is 9(*IQR*[7.5,10]) and for block size eight is 8(*IQR*[8, 16]). The possibility of stopping after a small number of participants is addressed in the Discussion section.

### 3.2 Misspecification of response rates

Misspecification of the response rate on treatment and/or control can lead to a probability of rejecting *H*_0_ that is considerably greater or lower than expected. The consequences of such misspecification may depend on the design approach used.

**Figure 4:**
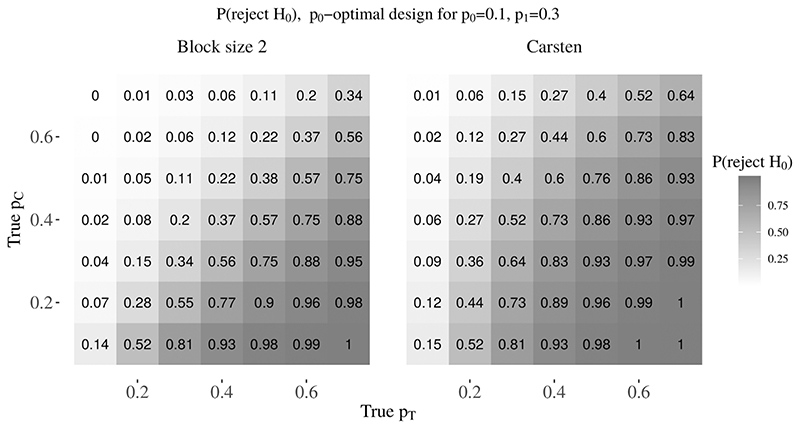
Probability of rejecting H0 when (p0, p1) are misspecified, for the p0-optimal Carsten design (r1, n1, r, Narm)= (1, 3, 7, 16) and block size two design (r, Narm, θF, θE) = (3, 31, 0.128, 0.932) under (α, β, p0, p1) = (0.15, 0.2, 0.1, 0.3)

**Figure 5:**
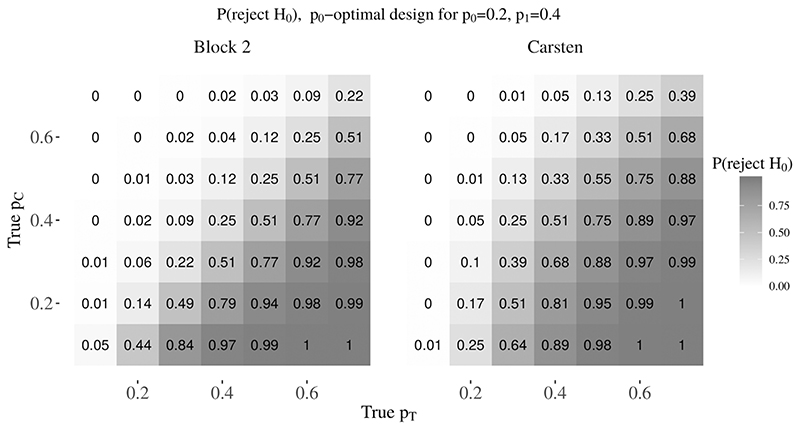
Probability of rejecting H0 when (p0, p1) are misspecified, for the p0-optimal Carsten design (r1, n1, r, Narm)= (2, 7, 10, 29) and block size two design (r, Narm, θF, θE) = (5, 48, 0.115, 0.964) under (α, β, p0, p1) = (0.15, 0.2, 0.2, 0.4)

Figure 4 shows the probability of rejecting *H*_0_ when the response rates (*p*_0_, *p*_1_) are misspecified, for the *p*_0_-optimal Carsten design and the block design with block size two under (*α*, *β*, *p*_0_, *p*_1_) = (0.15,0.20,0.10,0.30). The probability of rejecting H_0_ when *p_T_* > *p_C_* is given in the lower right triangles, while the probability of rejecting *H*_0_ when *p_T_* < *p_C_* is given in the upper left triangles. The probability of rejecting *H*_0_ when *p_T_* = *p_C_* is given by the remaining diagonal. Figure 5 shows the analogous probability of rejecting *H*_0_ under (*α*, *β*, *p*_0_, *p*_1_) = (0.15,0.20,0.20,0.40). The probabilities were obtained using simulations ofsize 10 000. In all instances, the probability of rejecting *H*_0_ is greater using the Carsten design than the block design with block size two. This difference is considerable for a number of plausible pairs of response rates. For example, in Figure 4, where the anticipated response rates are (*p*_0_, *p*_1_) = (0.1,0.3), P(reject *H*_0_) = 0.64 using the Carsten design (vs 0.34 using the block design) when (*p_C_*, *p_T_*) = (0.3,0.3) and P(reject *H*_0_) = 0.36 (vs 0.15) when (*p_C_*, *p_T_*) = (0.3,0.2). Similarly, in Figure 5, where the anticipated response rates are (*p*_0_, *p*_1_) = (0.2,0.4), P(reject *H*_0_) = 0.39 (vs 0.22) when (*p_C_*, *p_T_*) = (0.3,0.3), and P(reject *H*_0_) = 0.51 (vs 0.25) when (*p_C_*, *p_T_*) = (0.4,0.4). When using Carsten’s designs, if there is no difference between the treatment and control arms, and even if the treatment arm has a poorer response rate than the control arm, there may still be a substantial probability of rejecting *H*_0_ and concluding that the treatment response rate is of clinical interest. This is of particular concern as a key advantage of randomised trials over single-arm trials is greater accounting for response rate misspecification.^6^

### 3.3 Example trial

A trial that has been used previously as an example in comparing two-arm binary outcome trial designs is CALGB 50502, a randomised phase II trial for the treatment of Hodgkin Lymphoma.^20,22,33^ The design parameters of the trial are (*α*, *β*, *p*_0_, *p*_1_) = (0.15,0.20,0.70,0.85). Optimal designs for this set of design parameters were sought for the designs of Jung, Carsten and Chen, Chen et al and the block designs, using the same methods as for the main comparisons.^10,11,12^ The maximum sample size searched over was 200, with the exception of the Carsten and Chen design, where the maximum sample size was 400. However, no feasible designs were found using the Carsten and Chen design. Table 3 shows the *p*_0/1_-optimal and p_0/1_-minimax designs for the remaining designs. The *p*_0_-minimax and *p*_1_-minimax designs were again identical. The *p*_0_- and *p*_1_-optimal block designs reduce expected sample size by approximately one third compared to the existing designs, at the expense of increased maximum sample size. The maximum sample size for the p_0/1_- minimax designs are similar across all four two-arm designs, in the range [122,128], though here *ESS*(*p*_0_, *p*_0_) and *ESS* (*p*_0_, *p*_1_) are superior for the block designs compared to the existing designs. The Simon designs have expected sample sizes in the interval [20.7,30.2], while the standard two-arm trial with no early stopping has *ESS*(*N*)=*N* = 160. Again, *ESS*(*p*_0_, *p*_0_) and *ESS*(*p*_0_, *p*_1_) for both block designs is closer to the Simon design than the two-arm design, with the exception of the block size two design under *p*_0/1_-minimax.

## 4 Discussion

This manuscript introduces a new design for two-arm phase II binary outcome clinical trials. While continuous monitoring has previously been used in conjunction with NSC, this design is novel as it uses SC to reduce expected sample size. Curtailment may occur due to observing either a high or low response rate on the treatment arm compared to the control arm. Participants are allocated in a randomised block design, and trial results are noted after each block and compared to pre-specified stopping boundaries. The trial will end if the required final difference between the arm response rates is either certain to be reached or certain to not be reached. Additionally, the trial will end if the conditional power of reaching such a state is either greater than some upper threshold *θ_E_* or less than some lower threshold, *θ_F_*. These thresholds, in combination with the maximum sample size *N*, the required final difference in treatment arm response rates *r* and desired type I error and power determine the stopping boundaries.

The probability of rejecting the null hypothesis is controlled be at most *α* when *p_C_* = *p_T_* = *p*_0_ and at least 1 - *β* when *p_C_* = *p*_0_, *p_T_* = *p*_1_. However, if there is misspecification in the response rates, the probability of rejecting the null hypothesis may be affected. This has been addressed in Section 3.2. For the proposed designs, the type I error rate is maximised at *p_C_* = *p_T_* = 0.5, and so this error rate could be controlled over the interval [0, 1] by setting *p*_0_ = 0.5. However, this choice may not accurately reflect an investigator’s belief regarding the anticipated response rates.

The proposed block design was compared to three existing designs—those proposed by Jung, Carsten and Chen and Chen et al.^10,11,12^ All three designs include an interim analysis, while the designs of Carsten and Chen and Chen et al also use NSC.^11,12^ NSC is a way of permitting early stopping without the possibility of making a go/no go decision that is different to the decision that would be made if no early stopping was permitted. However, by also including an explicit interim analysis in addition to NSC, this certainty is somewhat negated, as a trial may stop early despite the possibility that a different decision may be made if the trial were to continue to completion, and the probability of such an occurrence is not quantified.^15^ Using the proposed design, this probability is quantified and maybe bounded if desired. A comparison between the proposed design and the three existing designs was undertaken using a loss function, a weighted sum of three optimality criteria. The type I error was set to *α* = 0.15 and power to 1 - *β* = 0.8, as in Table 1 of Jung.^10^ Five sets of response rates (*p*_0_, *p*_1_) were examined. For low values of *p*_0_, only the Carsten and Chen design was superior to the proposed block design. The superior performance of the Carsten and Chen design for low values of *p*_0_ has been previously noted by Chen et al.^12^ However, not discussed by Carsten and Chen nor Chen et al is sensitivity of the Carsten and Chen design to response rate misspecification. Such misspecification can lead to a considerable increase in the probability of rejecting *H*_0_ when the treatment is not sufficiently superior to control. For greater values of *p*_0_, the block design is superior to the compared designs for most combinations of weights, in terms of expected and maximum sample size, even when using blocks of size eight. Under the given requirements for type I error and power, the expected sample size of the design with block size eight is likely to be less than or approximately equal to that obtained using the designs of Carsten and Chen or Chen et al for *p*_0_ ≥ 0.3, and with the degree of monitoring reduced by a factor of four or eight, respectively.

The designs were also compared using a real-life example, used previously to compare two-arm designs.^10,22,33^ When minimising expected sample size under either *p_C_* = *p_T_* = *p*_0_ or *p_C_* = *p*_0_, *p_T_* = *p*_1_, the reduction in expected sample size for the proposed block designs was considerable compared to existing designs. When minimising maximum sample size, the proposed block designs had comparable maximum sample size and smaller expected sample size compared to existing designs, again with monitoring frequency reduced considerably when using blocks of size eight.

The designs of Carsten and Chen and Chen et al are examples of continuous monitoring, where, in contrast to the two-stage designs of Simon and Jung, the data are subject to more frequent interim analyses.^9,10,11,12^ When continuous monitoring is used, the number of interim analyses is dependent on the number of participants’ responses available, the point at which a stopping boundary is reached and the frequency of monitoring agreed. Monitoring may take place after every participant or less frequently.^16^ Continuous monitoring is of the greatest value when (tumour) response is measured over short periods of time, though it is possible to use curtailment and continuous monitoring for response time periods that may be considered long.^34^ Given the low recruitment rate of randomised controlled trials, for example, the median rate of0.92 participants per centre permonth reported in a review byWalters et al, the effect ofanylag on observed sample size is likely to be small.^35^ Further, trial recruitment rates are generally lower than expected, favouring more frequent monitoring.^15^

The designs of Jung, Carsten and Chen and Chen et al use what are known are binding stopping rules, whereby stopping is mandatory when any pre-specified stopping boundary is reached.^10,11,12^ This is in contrast to non-binding stopping rules, where, despite reaching a stopping boundary, a trial may continue for other reasons, for example, to gain more information regarding adverse events.^36^ Despite binding stopping rules being present, some trials have disregarded the planned stopping rules in practice using the same rationale as NSC, that is, the final decision is known with certainty due to the results so far. Such curtailment has been used both due to low and high observed response rates, and can only have been done by reviewing the results frequently.^34,37,38,39,40,41^ As such, some degree of continuous monitoring is being used in some trials where none is specified.

An advantage of using the block design over existing curtailed designs is that fewer interim analyses may be required. While Carsten and Chen’s design requires monitoring after every pair of participants and Chen et al’s design after every single participant, the degree of monitoring required for the block design depends on the block size used, and may be specified by the investigator.^11,12^ Further, use of larger blocks reduces computational burden with regards to the search for design realisations, with only a small increase in expected sample size.

Our proposed design would function similarly in practice to a group sequential design with many stages. As a comparison, it is possible to find group sequential designs of up to 10 stages using the rpact package in R.^42^ Using this software to find a design with the maximum number of stages and with the design parameters as specified as Table 2 results in a design with *ESS*(*p*_0_, *p*_0_) = 59.1, *ESS*(*p*_0_, *p*_1_) = 62.8 and *N* = 108 (code given in supplementary material). These results may be compared to the proposed approach using blocks of size eight (Table 2), which finds a *p*_0_-optimal design with *ESS*(*p*_0_, *p*_0_) = 49.2 (reduction of 17%), a *p*_1_-optimal design with *ESS*(*p*_0_, *p*_1_) = 49.3 (reduction of 21%) and a minimax design with *N* = 80 (reduction of 26%). The maximum number of stages in the *p*_0_- and *p*_1_-optimal cases would be 14 and in the minimax case would be 10, while using larger block sizes would result in fewer stages.

One limitation of the block design is that, depending on the design realisation, it is possible that a trial may end after as few as two participants if block size two is chosen, which some investigators may not be in favour of. However, among the set of five comparisons in the Results section, this did not occur for any of the four optimality criteria when block size two was used. Across these design realisations, the median minimum number of participants was found to be nine. Still, conservative investigators may prefer to either use a larger block size or to begin with a single large block (eg, a block of size 16) before switching to a smaller block size (eg, blocks of size four), guaranteeing a minimum number of participants equal to the size of the first block. In the latter case, this would require augmenting the existing code in order to obtain the trial operating characteristics. The block sizes used in practice may differ from the planned trial design. In this case, the stopping boundaries could be reassessed taking this into account. Further, stopping boundaries and conditional power could be re-estimated given the trial information so far. However, such extensions are beyond the scope of this article.

When using randomised blocks in single-centre trials, there may be concern that investigators may engage in selection bias by successfully “guessing” the arm to which the next participant will be assigned.^43^ This concern is attributable to simple block randomisation itself rather than the proposed trial design approach, but may still be briefly addressed. In the first instance, we assume that any randomised study is double-blinded, that is, investigators do not know which treatment is which.^7^ Selection bias may be further minimised by ensuring that the investigator responsible for selection does not take part in participant treatment assignment. Such steps may be taken independently of the design approach. Indeed, the CONSORT 2010 checklist of information to include when reporting a randomised trial includes “describing any steps taken to conceal the allocation sequence until interventions were assigned.”^44^ A further step that may be taken is to vary block sizes within a trial, though this would require an extension of the current work and again is beyond the scope of this article.

If a trial uses multiple centres and the randomised blocking is stratified by centre, then some imbalance may occur. Due to the typical size of phase II trials in oncology, we recommend using randomised blocks in a centralised system, that is, not stratified by centre, which would ensure balance.

Regarding the timing of interim analyses, it is the case that if a number of participant results emerge in quick succession then the interim analysis may not take place at the planned information fraction. Such a possibility has not negatively affected the popularity of the Simon design. As stated above, recruitment is often slow in clinical trials, with a median recruitment rate of approximately one patient per centre per month. This reduces the possibility of observing a cluster of concurrent results. Further, in some trials it is clear that investigators already monitor current results with the intention of stopping due to NSC, even when such analyses are not included in the planned design.^15^ A stopping boundary check should be undertaken as soon as results for each complete block are available. If this is somehow not possible and there exist excess results beyond a whole block, a stopping boundary check may still be undertaken using the results for participants whose results constitute completed blocks. This does not affect the operating characteristics of the design.

When using any clinical trial design that permits early stopping, the maximum likelihood estimator may be biased, though estimators have been developed that can be used for inference in such trials.^45^ Examples of estimators that may be used include the bias-adjusted estimator, the simplified bias-subtracted estimator, the median unbiased estimator (MUE) and the uniformly minimum-variance unbiased estimator (UMVUE).^46,47,48,49^ These estimators have been used in the group sequential, single arm setting to obtain point estimates and root mean squared errors.^15^

This article shows the benefit of using the proposed approach, which combines SC, randomised blocks and other features in a novel way. It provides the exact distribution of a trial’s outcomes, meaning that its operating characteristics are known without sampling error. Compared to other existing two-arm designs, the proposed approach considerably reduces expected sample size.

## Supplementary Material

Code

Readme file

## Figures and Tables

**Table 1:** Characteristics of the two-arm designs to be compared

Approach	NSC	SC	Early stopping for positive treatment effect	Early stopping without curtailment	No. stopping decisions	Test statistic
Jung	No	No	No	Yes	2	*X_T_* - *X_c_*
Carsten and Chen	Yes	No	Yes	Yes	*N*/2	$\sum I\left(X_{Ti}=1,X_{Ci}=0\right)$
Chen et al.	Yes	No	Yes	Yes	*N*	*X_T_* - *X_c_*
Block design	Yes	Yes	Yes	No	*N*/2*B*	*X_T_* - *X_c_*

*Note*: NSC: non-stochastic curtailment; SC: stochastic curtailment; *i* =1,2,…, *m*, where *m* is the number of participants per arm so far; *B*: number of participants per arm per block.

**Table 2:** *p*_0_-optimal, *p*_1_-optimal and *p*_0/1_-minimax designs, for (*α*, *β*, *p*_0_, *p*_1_) = (0.15,0.20,0.30,0.50)

	*r*_1_	*n*_1_	*r*	*N_arm_*	N	*ESS*(*p*_0_, *p*_0_)	*ESS*(*p*_0_, *p*_1_)	*θ_F_*	*θ_E_*
**p_0_-optimal**									
Simon	2	8	10	28	28	17.0	25.1	—	—
Jung	0	22	5	46	92	64.9	86.7	—	—
Carsten	5	19	12	44	88	51.3	60.3	0.000	1.000
Chen	0	16	5	45	90	60.1	76.9	0.000	1.000
Block 2	—	—	5	58	116	47.3	47.2	0.1348	0.9831
Block 8	—	—	5	56	112	49.2	49.3	0.3005	0.9700
**p_1_-optimal**									
Simon	3	13	8	21	21	17.6	20.6	—	—
Jung	-1	28	5	41	82	70.5	80.8	—	—
Carsten	9	30	10	46	92	55.0	52.6	0.000	1.000
Chen	4	35	4	38	76	63.3	67.3	0.000	1.000
Block 2	—	—	6	56	112	47.9	45.4	0.1072	0.9740
Block 8	—	—	5	56	112	49.2	49.3	0.3005	0.9700
**p_0/1_-minimax**									
Simon	3	13	8	21	21	17.6	20.6	—	—
Jung	-1	28	5	41	82	70.5	80.8	—	—
Carsten	4	20	10	34	68	53.3	52.9	0.000	1.000
Chen	4	35	4	38	76	63.3	67.3	0.000	1.000
Block 2	—	—	4	40	80	57.3	52.7	0.0428	0.9842
Block 8	—	—	4	40	80	62.2	57.1	0.0609	0.9752

*Note*: *N_arm_*: number of participants per arm.

**Table 3:** *p*_0_-optimal, *p*_1_-optimal and *p*_0/1_-minimax designs, for (*α*, *β*, *p*_0_, *p*_1_)=(0.15,0.20,0.70,0.85)

	*r*_1_	*n*_1_	*r*	*N_arm_*	N	*ESS*(*p*_0_, *p*_0_)	*ESS*(*p*_0_, *p*_1_)	*θ_F_*	*θ_E_*
**p_0_-optimal**									
Simon	10	14	25	33	33	20.7	30.2	—	—
Jung	-1	27	6	73	146	94.6	135.1	—	—
Chen	0	23	6	72	144	93.8	124.6	0.000	1.000
Block 2	—	—	6	99	198	61.1	79.4	0.1108	0.9928
Block 8	—	—	4	88	176	64.4	87.7	0.3391	0.9965
**p_1_-optimal**									
Simon	4	7	23	30	30	21.9	28.3	—	—
Jung	3	56	5	62	124	114.1	121.8	—	—
Chen	1	49	6	61	122	102.9	113.2	0.000	1.000
Block 2	—	—	6	99	198	61.1	79.4	0.1108	0.9928
Block 8	—	—	6	92	184	66.4	83.5	0.2730	0.9866
**p_0/1_-minimax**									
Simon	20	26	22	29	29	26.5	28.4	—	—
Jung	3	56	5	62	124	114.1	121.8	—	—
Chen	0	46	6	61	122	102.1	113.4	0.000	1.000
Block 2	—	—	5	62	124	96.4	95.9	0.0064	0.9960
Block 8	—	—	5	64	128	80.1	91.7	0.1304	0.9887

*Note*: *N_arm_*: number of participants per arm.

## Data Availability

The code to undertake the design search for the proposed design was written in R, and is available in the supplementary material and on github.^32^
