# Supplementary material for: A stochastically curtailed two-arm randomised phase II trial design for binary outcomes
Source: Pharm Stat. Author manuscript; Available in PMC 2022 Jan 5. (PMC7612167; doi:10.1002/pst.2067)
Supplement: Readme file [file EMS140635-supplement-Readme_file.pdf]

README file for: A stochastically curtailed two-arm randomised phase II trial design for binary outcomes

Martin Law, Michael J. Grayling, Adrian P. Mander

August 5, 2020

The supplementary material consists of six files, detailed below. All files and code can be found online at `https://github.com/martinlaw`.

- `examples.R`: Examples of how to find stochastically curtailed designs. Uses functions from the file `1-reproduce-results-functions-find-designs.R`.

- `1-reproduce-results-functions-find-designs.R`: Functions that can be used to find the designs used in the manuscript, and by any user to find stochastically curtailed designs.

- `2-reproduce-results-find-designs.R`: This code finds the designs used in the manuscript. Depending on computing power, this may take days. As an alternative, one may simply load the file `data.RData`. An alternative is to decrease the value of *max.combns* from $1e6$ to $1e5$ or even $1e4$, though this may result in poorer designs.

- `data.RData`: A file containing the results of `2-reproduce-results-find-designs.R`.

- `3-reproduce-results-functions-for-tables-plots.R`: Functions used to create the tables and plots in the manuscript.

- `4-reproduce-results-tables-plots.R`: This code creates the tables and plots found in the manuscript.
